# Instance Segmentation of Multiple Myeloma Cells Using Deep-Wise Data Augmentation and Mask R-CNN

**DOI:** 10.3390/e24010134

**Published:** 2022-01-17

**Authors:** May Phu Paing, Adna Sento, Toan Huy Bui, Chuchart Pintavirooj

**Affiliations:** 1 School of Engineering, King Mongkut’s Institute of Technology Ladkrabang, Bangkok 10520, Thailand; 2 Faculty of Engineering, Thai-Nichi Institute of Technology, Bangkok 10250, Thailand; adna@tni.ac.th; 3 Course of Science and Technology, Graduate School of Science and Technology, Tokai University, Tokyo 108-8619, Japan; 9ltad006@mail.u-tokai.ac.jp

**Keywords:** multiple myeloma, plasma cells, deep learning, Mask R-CNN, data augmentation

## Abstract

Multiple myeloma is a condition of cancer in the bone marrow that can lead to dysfunction of the body and fatal expression in the patient. Manual microscopic analysis of abnormal plasma cells, also known as multiple myeloma cells, is one of the most commonly used diagnostic methods for multiple myeloma. However, as it is a manual process, it consumes too much effort and time. Besides, it has a higher chance of human errors. This paper presents a computer-aided detection and segmentation of myeloma cells from microscopic images of the bone marrow aspiration. Two major contributions are presented in this paper. First, different Mask R-CNN models using different images, including original microscopic images, contrast-enhanced images and stained cell images, are developed to perform instance segmentation of multiple myeloma cells. As a second contribution, a deep-wise augmentation, a deep learning-based data augmentation method, is applied to increase the performance of Mask R-CNN models. Based on the experimental findings, the Mask R-CNN model using contrast-enhanced images combined with the proposed deep-wise data augmentation provides a superior performance compared to other models. It achieves a mean precision of 0.9973, mean recall of 0.8631, and mean intersection over union (IOU) of 0.9062.

## 1. Introduction

Multiple myeloma (MM)—*Commonly* known as bone malignancy—is the proliferation of malignant plasma cells within the bone marrow. Bone marrow is a soft, spongy tissue found in human bones and is made of three different cells, namely red blood cells (RBCs), white blood cells (WBCs) and platelets [1]. Approximately eighty to ninety percent of the bone marrow is filled with WBCs—essential cells for the human body’s immune system. More precisely, B lymphocytes or B cells, a type of WBCs, make antibodies to fight against infections and maintain humoral immunity. Once B cells respond to infections, they mature and change into plasma cells. In healthy conditions, plasma cells produce antibodies called “immunoglobulin” to fight against infections and diseases [2]. However, when myeloma cancer occurs, plasma cells in the bone marrow accumulate and crowd out other healthy RBCs and platelets. The main cause of myeloma is due to the deoxyribonucleic acid (DNA) damages or changes during new plasma cell production [1]. In medical terms, those produced abnormal plasma cells are called myeloma cells. Instead of producing normal antibodies, the myeloma cells produce monoclonal antibodies that can lead to bone problems, especially damage to the bone marrow and brittle bones. Unlike other types of cancer, myeloma cancer will not form a tumor or a lump. However, it can affect other parts of the body; hence it is called multiple myeloma (MM). Some examples of complications caused by MM include renal failure, frequent infections, improper kidney functions, and low red blood cell count (anemia). 

Based on the statistical data of the Global Cancer Observatory (GLOBOCAN), approximately 160,000 global incidences of MM were found in 2018, and it was 0.9% of all cancer diagnoses [2]. Moreover, there were around 106,000 global mortality cases, approximately 1.1% of all cancer deaths. Common symptoms of MM include bone pain, especially in the spine or chest, nausea, constipation, loss of appetite, mental fogginess or confusion, fatigue, frequent infections, weight loss, weakness or numbness in legs, and excessive thirst. When a patient suffers symptoms that are suspected to be MM, he or she needs to perform one or more diagnostic tests, for example, (i) lab tests such as complete blood count (C.B.C.), blood chemistry test, urine test, quantitative immunoglobulins, electrophoresis, serum-free light chains; (ii) bone imaging tests, such as x-rays, computed tomography scans, magnetic resonance imaging (MRI), positron emission tomography (PET) scans; and (iii) biopsies such as bone marrow biopsy and aspiration, fine-needle aspiration biopsy, and core needle biopsy. Among those diagnostic tests, our research will focus on bone marrow aspiration. Generally, biopsy and the aspiration of bone marrow have quite similar procedures. Bone marrow consists of both solid and fluid parts. A bone marrow biopsy removes a small sample of solid tissue from the bone marrow inside the patient’s bones for testing. It is unlikely that aspiration takes the fluid sample from the bone marrow. The collected samples are then lysed onto a slide and stained [3]. Thereafter, a microscopic exam of the stained slides is conducted to investigate the percentage of plasma cells in the bone marrow. 

For instance, a microscopic image captured from bone marrow aspirate slides of patients can be seen in the figures of Section 3.2.1. As we can see in the figures, the appearance of myeloma cells and normal plasma cells is very similar; thus, it is very complex to differentiate cells from each other. Generally, histology and morphological features of the cells are needed to be thoroughly analyzed to differentiate them [1]. Therefore, in clinical practice, one or more expert pathologists manually analyze the stained slides from the microscopic images to detect the MM cells. However, as it is a manual process, it has many limitations, such as being effort-and-time consuming as well as being sensitive to human errors. For those reasons, researchers in computer and technological fields tried to create computer-aided tools for a more straightforward, faster, and more reliable diagnosis of MM. 

Many research papers focused on the automatic detection and segmentation of cells from microscopic images, but, to the best of our knowledge, there are inconsiderable numbers of papers that emphasize myeloma cells. The automatic detection and segmentation of plasma cell segmentation for myeloma diagnosis is very challenging and complicated due to the following reasons [4]:

(1) The number of nucleus and cytoplasm may vary from one cell to another;
(2) The boundary of the plasma cells is fuzzy because the cytoplasm of the cell and the background of the image have a similar visual appearance;
(3) In some situations, cells may be isolated as single cells, but sometimes they may be in clusters;
(4) If cells are in clusters, they may be in three different conditions: (i) Nuclei of cells are touching; (ii) Cytoplasm of cells are touching; and (iii) Cytoplasm of one cell is touching the nucleus of other cells. In such cases, the computer-aided segmentation process will be more challenging because the cytoplasm and nucleus expose different colors;
(5) In some situations, it is possible to have an unstained cell (for example, a red blood cell) underneath the cell of interest (MM plasma cell). In such cases, the color and shade of unstained cells may change and interfere with MM plasma cells. As a negative result, it may interfere with the detection and segmentation of cells of interest.

Therefore, resolving the aforementioned difficulties is very important in developing a high-performance computer-aided diagnosis system.

## 2. Related Works

The literature on computer-assisted diagnostic tools for multiple myeloma can be roughly divided into two main parts, namely: (i) conventional machine learning-based methods; and (ii) modern deep learning-based methods. Significantly, research conducted in the 2010s applied image processing, feature extraction, and conventional machine learning algorithms. For instance, Saeedizadeh et al. [5] (2015) applied contrast enhancement for image preprocessing and thresholding for nucleus and cytoplasm detection. Moreover, they applied bottleneck and watershed methods for separating touching cells. Then, features such as nucleus eccentricity and nucleus–cell ratio were extracted and classified using a support vector machine (SVM) classifier. They utilized a local dataset that contained 50 images with 678 cells (132 normal, 256 myelomas, and 290 other). They reported a sensitivity of 96.52%, a specificity of 93.04% and a precision of 95.28%, respectively. Similarly, in 2018, another conventional machine-learning-based MM diagnosis was proposed by Gupta et. al. [4]. Their method was called PCSeg, and it mainly applied multi-phased level set theory. Similar to [5], PCSeg also used watershed and circular Hough transform methods for post-processing after segmentation. The performance of PCSeg was evaluated using a public dataset called the MiMM_SBILab Dataset [6], which contains 85 microscopic cell images, and it achieved 81.66% of recall, 76.56% precision, and 79.03% of F1-scores. Even though the conventional machine learning-based methods reported high performance, the outputs of those methods are highly dependent on the prior information of the images or manually predefined parameters.

With the recent and advanced improvements in technology, deep learning-based computer-assist tools became state-of-the-art systems. For example, a deep neural network-based myeloma cell detection was found in Tehsin et al. [7]. They applied AlexNet as a feature extractor to extract deep features from microscopic cells and then classified them using SVM. Before extracting the features, they analyzed and preprocessed the images based on a particular color channel. They used the same dataset as [5], containing 50 microscopic images (34 with plasma cells and 16 with normal cells). They reported 89% sensitivity without preprocessing and 100% with preprocessing. Nonetheless, their method performed the classification only, and it could not provide the specific locations of the plasma cells.

Moreover, Vishnav et al. [3] also performed MM cell segmentation by comparing two deep neural networks such as Mask R-CNN and U-Net. They validated their proposed method on the MiMM_SBILab dataset [6]. They observed that Mask R-CNN (with a precision of 82.61%) outperformed the U-Net (with a precision of 78.95%). However, in the study of Voula et al. [8], U-Net gave a better performance in nucleus segmentation than Mask R-CNN in terms of similarity index criteria. Meanwhile, Mask R-CNN was better if the precision criteria were used. Therefore, they finally decided to develop an ensemble of Mask R-CNN and U-Net to get the best outputs. Their approach provided a promising result (0.523 mAP and 0.725 precision) on an extensive dataset containing 800 images with different modalities, staining and microscopy techniques. Nevertheless, their focus was solely on the segmentation of cell nuclei.

Bozorgpour et al. [1] also proposed an automatic myeloma cell detection using a two-staged deep learning framework. First, they segmented the nucleus using U-Net at the initial stage. Then, as the second stage, the cytoplasm of cells was segmented using multi-scale regional attention deeplab3+. They also proposed an aggregation function to select the most related scale for a single prediction. Their proposed method was the second winner of the SegPC2021 competition and achieved a mean IOU value of 0.9385.

Unlike the previous studies, our paper performs multiple myeloma detection with two main contributions. The first contribution is developing different Mask R-CNN models not only using original microscopic images but also using contrast-enhanced and stained cell images. The main reason behind the first contribution is due to the wide variability natures of MM cells. As discussed in the introduction, such variability makes the computer-aided detection and segmentation of MM cells very challenging. Therefore, we used contrast enhancement and stained cell images to increase the separability between myeloma cells and non-myeloma regions. Moreover, as a second contribution, we propose a deep learning-based data augmentation method called deep-wise augmentation to enhance the performance of Mask R-CNN models. The details of these contributions will be discussed in the later sessions. The rest of the paper is organized as follows: Section 3 will discuss the materials and methods applied in this paper. Then, Section 4 will discuss the procedures of experiments to validate the proposed method and discuss the experimental findings. Finally, Section 5 will conclude the paper.

## 3. Materials and Methods

### 3.1. Materials 

The microscopic images of multiple myeloma cells applied in this research were obtained from the TCIA_SegPC_dataset [4,9,10]. It is a publicly released dataset for academic and research purposes after the SegPC2021 challenge. It contains 775 images in total, divided into three parts: training set (298 images), validation (200 images) and testing (277 images). The ground truth data for the myeloma cells in the training and validation dataset are provided along with the dataset. We apply 80% and 20% of the training set for training and validation. The validation set from the original dataset is used for testing since the ground truth data for the original test set is not available. The images in the dataset are 24 bits bitmap images (.bmp) with dimensions of 2560 × 1920.

### 3.2. Methods

**Figure 1:**
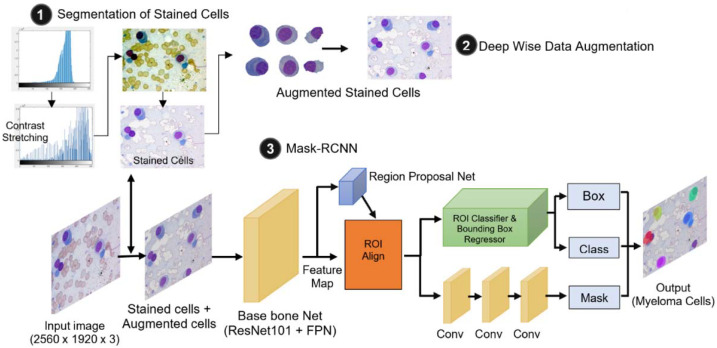
Semantic diagram of proposed multiple myeloma cell detection. It contains three major parts: (1) Segmentation of stained cells, (2) Deep-wise data augmentation, and (3) Mask R-CNN.

The semantic diagram of the proposed MM detection system is illustrated in Figure 1. As denoted in the figure, it consists of three major parts: (1) segmentation of stained cells; (2) deep-wise data augmentation; and (3) Mask R-CNN. The first part—stained cell segmentation—is a preprocessing task that enhances the quality of the input images. It aims to improve the learning ability of Mask R-CNN by removing the unfocused regions (unstained cells) from the input images. Then the second part—deep-wise augmentation—generates augmented images of stained cells as naturally as possible. Finally, the last part—Mask R-CNN—conducts the instance segmentation of myeloma cells. The details of each part are discussed in the following sub-sections. 

#### 3.2.1. Segmentation of Stained Cells 

**Figure 2:**
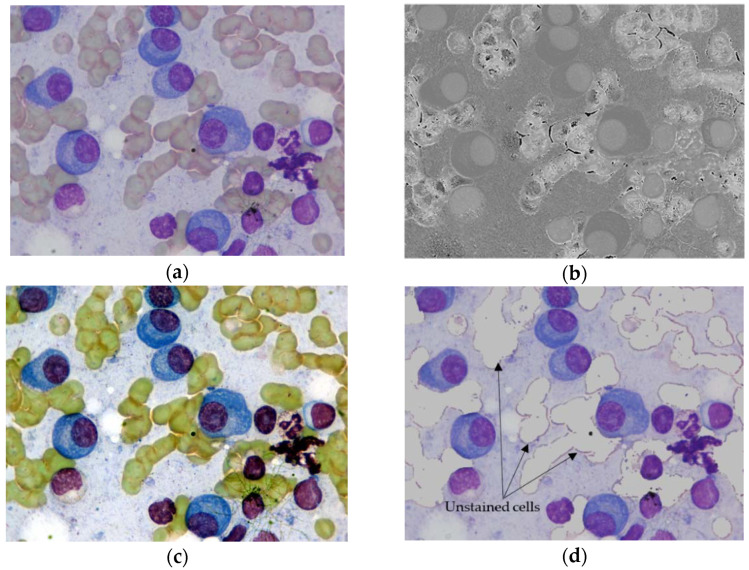
Segmentation of stained cells. (**a**) Original input microscopic cell image, (**b**) Hue (H) color channel of input image, (**c**) Result of contrast stretching, and (**d**) Result after removing unstained cells.

Multiple myeloma cells in their natural states are indistinguishable from normal cells. Staining is an essential procedure in microbiology since it enhances the contrast and visualization of cells under a microscope. The microscopic images used in this research had already undergone a stain color normalization. For example, Figure 2a demonstrates an input microscopic cell image. The stained objects in the figure generally expose blueish color, whereas unstained parts expose a pinkish color. We try to remove unstained cells because they can hinder the learning process of Mask R-CNN. A study by Gupta [4] performed a detailed analysis of different objects from the input cells images. They emphasized four different objects (ROIs): the nucleus of plasma cells, cytoplasm of plasma cells, unstained cells, and background. The separability of those ROIs was then analyzed in different color spaces, namely, RGB, HSV, and Lab. They reported that the maximum separability between stained and unstained cells could be obtained in the H color channel. 

However, in this study, we used the contrast stretching method to remove the unstained cells because it can provide better separability compared to the H channel images. Contrast stretching can improve the quality of the images by stretching a specific range of intensity values to fill the entire dynamic range [11]. As we can notice in Figure 2c, the result of contrast stretching turns the color of unstained regions into greenish, making them easier to distinguish from stained cells and background. Note that, in our ablation study (Section 4.1), we will develop Mask R-CNN using the contrast stretched images to validate its improvements in the separability of cells and learning abilities of Mask R-CNN models. After enhancing the contrast of the original input images, we remove unstained cells by replacing greenish color pixels with the background pixels, as illustrated in Figure 2d. Instead of deleting or changing the unstained pixels into zeroes, replacing them with background pixels ensures no additional regions for segmentation. 

#### 3.2.2. Deep Wise Data Augmentation

Advances in deep learning methods provided significant improvements in computer-aided diagnosis. Nevertheless, the performance of deep learning models crucially depends on the quality and sufficiency of input data. In practice, a common problem of computer-aided diagnosis methods is a shortage or a limited number of ground-truth data. Acquiring high-quality ground-truth data is very cumbersome because it is a manual process; thus, it is time-consuming, human-intensive, and sometimes due to the privacy of the medical data. Data augmentation, a process of creating new data by transforming or synthesizing existing data, is the most widely used solution for data insufficiency. It introduces the variability of the images in the dataset and improves the generalization capabilities of the learning model. It also works as a regularizer and solves the overfitting problem. The most commonly used data augmentation methods for current deep learning models include cropping, flipping, rotation, translation, color augmentation, and so forth. Selecting appropriate augmentation methods is oriented to the nature of images applied, the amount of data in the dataset. Moreover, it may also rely on the main objective of using the deep learning model and the expected performance. 

The specific objective of this research is to perform instance segmentation of myeloma cells from microscopic images. Once we compare the number of myeloma cells to the non-myeloma regions, it is found that the myeloma cells dataset suffers an imbalance problem. That is, the number of pixels in the myeloma cell regions is relatively fewer than those of other regions. Although data augmentation can solve the overfitting, it can affect the balance of the dataset as a trade-off. It will force an increase in the number of images by generating new images of both minority (myeloma cells) and majority classes (non-myeloma regions). For this reason, in this paper, we propose deep-wise data augmentation that increases the minority class while controlling the augmentation of the majority class. 

**Figure 3:**
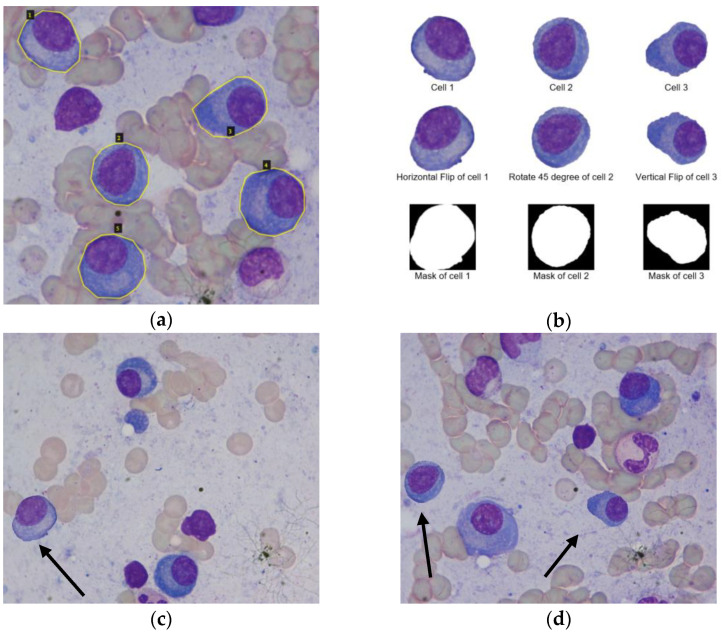
Deep-wise data augmentation. (**a**) Example of polygon masks (ground truths) for myeloma cells using VGG annotation tool, (**b**) Basic data augmentations of cells 1, 2, and 3, (**c**) Deep-wise augmentation of cell 1 (denoted by arrow), and (**d**) Deep-wise augmentation of cell 2 and 3 (denoted by arrows).

Initially, all myeloma cells from the training dataset are extracted using the polygon masks created for Mask R-CNN. As an illustrative example, Figure 3a shows polygonal masks of five myeloma cells of an input image from the training dataset. These masks are generated using the VGG image annotator [12], and they will be used to train Mask R-CNN. Then, Figure 3b shows the extracted myeloma cells (cell 1, 2, and 3) using polygon masks. Subsequently, the basic augmentation methods, such as the flipping and rotation of extracted myeloma cells are performed. Note that these data augmentation methods are not applied for the whole input images. They are only applied for myeloma cell regions (minority class); thus, it can prevent the increase in the number of pixels in non-myeloma regions (majority class). 

As we discussed earlier, the ultimate goal of our data augmentation is to augment the minority class objects (myeloma cell regions) while limiting the increase of majority class objects (non-myeloma regions). Based on Figure 3, we can increase the number of myeloma cells, and it seems like we have reached our goal. Nevertheless, the augmented myeloma cells were isolated as they were derived from the extracted cells using polygon masks. The real nature of cells is sometimes in a cluster, as we discussed in the introduction. Therefore, our augmented myeloma cells are not very realistic yet. Besides, the dimensions of augmented cells vary among cells and are different from the dimension of the input image. The possible solution to those problems is pasting the augmented cells on the background image containing other non-myeloma regions. 

Our proposed deep-wise data augmentation method will try to generate augmented myeloma cells as realistically and naturally as possible. Instead of directly pasting the augmented cells onto the background, it applies the deep learning-based blending method, inspired by deep image blending by Zhang et al. [13,14]. It blends the foreground objects, augmented myeloma cells, with the background in order to get more realistic effects. Unlike the original deep blending method [13], our proposed deep-wise data augmentation blends only the edge regions of the augmented cells and keeps remain the pixel values inside the cells the same. Because we do not want to lose or change the pathological and morphological information of pixels inside the myeloma cell regions. Moreover, to solve the problem of increasing the majority classes (non-myeloma regions), we randomly select the background images from the existing images of the training dataset. The random, augmented cells are composited and blended onto the selected background. Figure 3c illustrates the augmented images generated by our proposed deep-wise augmentation method. 

#### 3.2.3. Mask R-CNN 

Mask R-CNN [15] is one of the most famous cutting-edge algorithms for instance segmentation. It works on a two-staged structure. The first stage is a region proposal network (RPN) that generates the proposals of the regions that might be our region of interest (myeloma cells). The second stage is an R-CNN detector that predicts the class of that object and generates the bounding boxes and binary masks. The architecture of the proposed Mask R-CNN can be seen in the semantic diagram (Figure 1). The input images to the first layer of Mask R-CNN are images of three types: original images, contrast-enhanced images, stained cell images, and their associated polygon masks. We use ResNet101 and Feature Pyramid Network (FPN) as a backbone of the proposed Mask R-CNN. ResNet101 produces a feature map containing the low-level and high-level features from the inputs. Then, the produced feature map is improved by FPN. Using the improved feature map, the Region Proposal Network (RPN) will find all possible areas that contain myeloma cells. Subsequently, ROI Align part will refine the locations and sizes of ROIs proposed by the RPN. Afterwards, the specific classes of the ROIs (i.e., binary classes of myeloma or non-myeloma cells) and their associated bounding boxes are generated through the ROI classifier and bounding box regressor. Finally, a convolutional network will generate the segmentation masks of ROIs by taking the positive regions selected by the ROI classifier. 

Based on the literature review, the outperformance of Mask R-CNN for MM detection had been reported in [1,3,8]. However, all those methods applied the Mask R-CNN directly from the original input images. Our Mask R-CNN will differ from previous studies in two ways: (i) using three types of images: original image, contrast-enhanced images, and stained cell images; (ii) adding more augmented cells using deep-wised augmentation method. We will analyze, compare, and contrast the effectiveness of each image type in the following experimental results and discussion session. 

## 4. Experimental Results and Discussion

The most initial and essential part of the experiments is data preparation. As discussed in the materials (Section 3.1), we divide the original dataset into three parts—training, validation, and testing. The training part is used to develop Mask R-CNN models, and the validation part is for hyper-parameter tuning. We developed nine different Mask R-CNN models: (i) Original Mask R-CNN that uses original microscopic images; (ii) Original Augmented Mask R-CNN that uses original microscopic images with basic data augmentation methods; (iii) Original Mask R-CNN with Deep-wise data augmentation that uses original images and deep-wise augmented cells; (iv) Contrast-Enhanced Mask R-CNN that uses contrast-enhanced images; (v) Contrast-Enhanced Augmented Mask R-CNN that uses contrast-enhanced images with basic data augmentation methods; (vi) Contrast-enhanced Mask R-CNN with deep-wise data augmentation; (vii) Stained Cells Mask R-CNN that uses the images with only stained cells; (viii) Stained Cells Augmented Mask R-CNN that uses the images with only stained cells and basic augmentation methods; and (ix) Stained Cells Mask R-CNN with deep-wised data augmentation. All models have trained 200 epochs on an Nvidia GeForce RTX 3080 and saved the models that provide the highest validation performance. 

### 4.1. Ablation Study

The ablation study aims to analyze the performance of our proposed method in a step-by-step procedure, and it will help to find a Mask R-CNN model with the highest performance. We apply three assessment measures, namely mean precision, mean recall and mean IOU, to identify the performance of the models. Table 1 summarizes and compares the results of all models. 

By analyzing the assessment measures in Table 1, we can reveal that using contrast-enhanced images can provide a higher performance compared to the original and stained cell images. We expected that the performance of using stain cell images would be better but, in reality, the performance is not as good as contrast-enhanced images. Moreover, we found that basic data augmentation can increase the performances of models in all image types (i.e., original, contrast-enhanced, and stained cell images). It can increase approximately up to 0.0586 in mean precision, 0.0406 in mean recall and 0.0725 in mean IOU for all image types. For our proposed deep-wise data augmentation, it can provide the highest performance in all models. Compared to no augmentations, deep-wise augmentation can increase the performance of models approximately up to 0.0648 in mean precision, 0.0518 in mean recall and 0.1213 in mean IOU, respectively.

**Figure 4:**
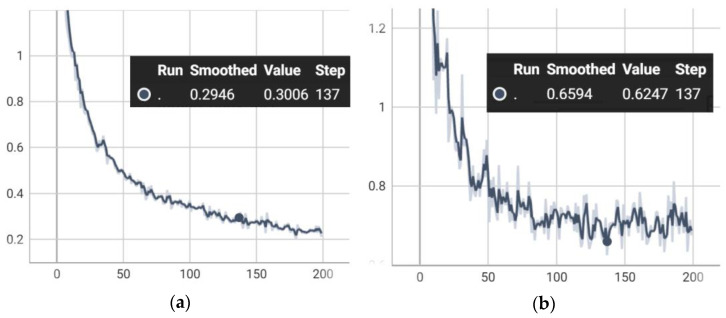
Training and validation of proposed Contrast-enhanced Mask R-CNN with Deep-wise data augmentation. (**a**) Training loss (0.3006) and (**b**) Validation loss (0.6247).

Therefore, we can summarize that, among all models, contrast-enhanced Mask R-CNN with deep-wise data augmentation provides the highest performance with a mean precision of 0.9973, mean recall of 0.8631, and mean IOU of 0.9062. It reached the highest performance with a training loss of 0.3006 and a validation loss of 0.6247 at epoch 137, as illustrated in Figure 4. 

**Figure 5:**
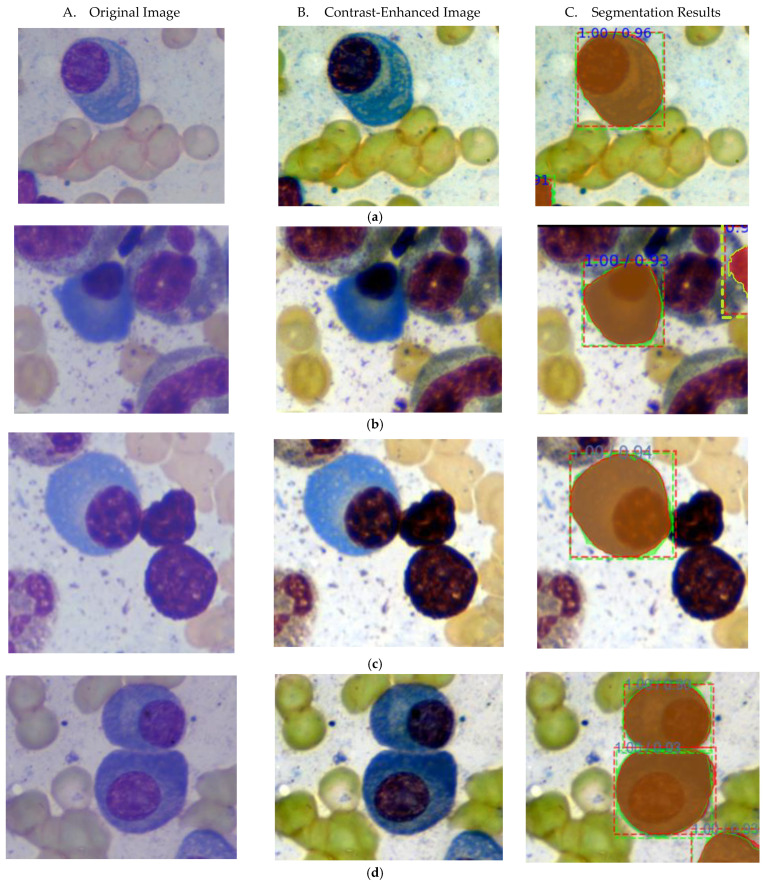
Some examples of segmentation results, by contrast-enhanced Mask R-CNN with deep-wise data augmentation. Each column represents the original image, contrast-enhanced image and segmentation result, respectively. (**a**) Isolated MM cell (**b**) Nucleus and cytoplasm have similar color intensity (**c**) MM cells in a cluster (Touching nuclei) (**d**) MM cell in a cluster (Touching cytoplasm), Green colored bounding boxes and masks represent ground truth, and the red colored ones represent segmentation result. The caption above the bounding box shows the prediction score/IOU value.

Some examples of segmentation results using our proposed method are illustrated in Figure 5. From this figure, it is evident that our proposed method has promise to resolve the challenges that are commonly found in automatic MM detection. Figure 5a shows the segmentation of isolated MM cells. The green color bounding box and mask represent the ground truth, and the red ones represent the outputs of our proposed method. The caption above the bounding box describes the prediction score and IOU value for each segmentation result. The MM cell in Figure 5a achieved a prediction score of 1.00 with an IOU value of 0.96. Similarly, Figure 5b shows an example of an MM cell having a very similar color intensity to a nucleus. Our proposed method can segment it with a prediction score of 1.00 with an IOU of 0.93. Moreover, the segmentation results of cluster cells are also demonstrated in Figure 5c,d. Figure 5c represents cluster MM cells with touching nuclei and Figure 5d represents clusters with touching cytoplasm, respectively. For a cluster with touching nuclei in Figure 5c, we got a precision score of 1.00 and an IOU of 0.94. For a cluster with touching cytoplasm in Figure 5d, it can successfully separate the touching boundary and well segment both cells with high IOU values 0.90 and 0.92.

### 4.2. Comparison with State-of-Art Methods

Among a number of previous methods described in the literature review, we select Mask R-CNN [3], U-Net [3], and U-Net plus Attention deeplab3+ [1] for performance comparison because those methods are based on deep learning methods with the same objective. Table 2 summarizes the results of the performance comparison. Based on these results, we can reveal that our proposed method achieved higher precision and IOU than Mask R-CNN [3] and U-Net [3], where the IOU of those methods are calculated from their reported confusion matrix [3]. Our proposed method is a slightly lower IOU (0.03) compared to deeplab3+ [1]. Nevertheless, our method would be a favorable alternative that can provide promising results with high precision (0.9973) while using a single-staged detection.

## 5. Conclusions

In this study, we proposed an automatic detection and instance segmentation of multiple myeloma cells from microscopic images of bone marrow aspiration slides. We developed Mask R-CNN models using microscopic images taken from bone marrow aspiration. We built nine different Mask R-CNN models using original, contrast-enhanced, and stained cell images. Moreover, we also applied deep-wise data augmentation to improve the performance of Mask R-CNN models. The models were developed using a public dataset of microscopic myeloma cell images, and the performances of all models were compared. Based on the empirical findings, the Mask R-CNN using the contrast-enhanced images with deep-wise augmentation produced the best performance with a mean precision of 0.9973, a mean recall of 0.8631, and an IOU of 0.9062, respectively. Our proposed method well detects and segments not only the isolated but also a cluster of myeloma cells. For future work, the performance of our method can be further improved using an advanced modification of Mask R-CNN such as an attention Mask R-CNN or ensemble Mask R-CNNs.

## Figures and Tables

**Table 1:** Performance comparison of nine different Mask R-CNN models.

Model	Mean Precision	Mean Recall	Mean IOU
Original Mask R-CNN	0.8769	0.8178	0.7689
Original Augmented Mask R-CNN	0.9426	0.8406	0.7465
Original Mask R-CNN with Deep-wise data augmentation	0.9464	0.8478	0.8721
Contrast-enhanced Mask R-CNN	0.8966	0.8333	0.7324
Contrast-enhanced Augmented Mask R-CNN	0.9843	0.8566	0.8879
Contrast-enhanced Mask R-CNN with Deep-wise data augmentation	0.9973	0.8631	0.9062
Stained cell Mask R-CNN	0.9389	0.7372	0.6478
Stained cell Augmented Mask R-CNN	0.9614	0.8130	0.7324
Stained cell Mask R-CNN with Deep-wise data augmentation	0.9632	0.8328	0.7348

**Table 2:** Performance comparison with state-of-the-art methods.

Methods	Precision	IOU
Mask R-CNN [3]	0.8261	0.7755
U-Net [3]	0.7895	0.6122
U-Net, Attention deeplab3+ [1]	-	0.9385
Proposed Method	0.9973	0.9062

## Data Availability

The microscopic images of multiple myeloma cells applied in this study can be downloaded from TCIA_SegPC_dataset via https://ieee-dataport.org/open-access/segpc-2021-segmentation-multiple-myeloma-plasma-cells-microscopic-images (accessed on 19 December 2021).
